# Metabolism of Dendritic Cells in Tumor Microenvironment: For Immunotherapy

**DOI:** 10.3389/fimmu.2021.613492

**Published:** 2021-02-24

**Authors:** Xin Peng, Youe He, Jun Huang, Yongguang Tao, Shuang Liu

**Affiliations:** ^1^Department of Oncology, Xiangya Hospital, Central South University, Changsha, China; ^2^Department of Translational Medicine, Cancer Biological Treatment Center, Xiangya Hospital, Central South University, Changsha, China; ^3^Institute of Medical Sciences, Xiangya Hospital, Central South University, Changsha, China; ^4^Department of Neurosurgery, Xiangya Hospital, Central South University, Changsha, China; ^5^Key Laboratory of Carcinogenesis and Cancer Invasion, Ministry of Education, Department of Pathology, Xiangya Hospital, Central South University, Changsha, China; ^6^Key Laboratory of Carcinogenesis of Ministry of Health, Cancer Research Institute, School of Basic Medicine, Central South University, Changsha, China; ^7^Hunan Key Laboratory of Tumor Models and Individualized Medicine, Department of Thoracic Surgery, Second Xiangya Hospital, Central South University, Changsha, China; ^8^Department of Oncology, Institute of Medical Sciences, National Clinical Research Center for Geriatric Disorders, Xiangya Hospital, Central South University, Changsha, China

**Keywords:** dendritic cells, tumor microenvironment, metabolism, glucose, amino acid, lipid, therapy

## Abstract

Dendritic cells (DCs) are a type of an antigen-presenting cell which undertake a job on capturing antigens coming from pathogens or tumors and presenting to T cells for immune response. The metabolism of DCs controls its development, polarization, and maturation processes and provides energy support for its functions. However, the immune activity of DCs in tumor microenvironment (TME) is inhibited generally. Abnormal metabolism of tumor cells causes metabolic changes in TME, such as hyperglycolysis, lactate and lipid accumulation, acidification, tryptophan deprivation, which limit the function of DCs and lead to the occurrence of tumor immune escape. Combined metabolic regulation with immunotherapy can strengthen the ability of antigen-presentation and T cell activation of DCs, improve the existing anti-tumor therapy, and overcome the defects of DC-related therapies in the current stage, which has great potential in oncology therapy. Therefore, we reviewed the glucose, lipid, and amino acid metabolism of DCs, as well as the metabolic changes after being affected by TME. Together with the potential metabolic targets of DCs, possible anti-tumor therapeutic pathways were summarized.

## Introduction

There are three phases in the tumor formation process, which are immune surveillance, immune balance, and immune escape (1). Once tumor cells gain the ability to encounter the immune system, the next step is to nest and get used to the local environment and make its surroundings favorable to its further expansion (2). Gradually, tumor microenvironment (TME) is formed. There are three types of tumors in terms of the TME immune response status, which are inflammatory tumor or hot tumor, immune inhibitory tumor, and immune escape tumor or cold tumor (3). In a hot tumor, immune cells can act against a tumor cell and cause an inflammatory response at the local site. In the immune inhibitory tumor, inhibitory immune cells derived from bone marrow dominated in its TME and the adaptive immune response which started from dendritic cells (DCs) are inactive. In a cold tumor, immune cells cannot get inside of TME for the lack of major histocompatibility complex (MHC) I on tumor cells or no specific tumor-related antigen presented by MHC-I; in this case, immune therapy usually fails to achieve the expected response (4). Here, we seek to focus on the first two types of TME, specifically on a key component of the adaptive immune reaction chain, DCs. We also focus on how their metabolism status influences their function, what can done to regulate DCs to our purposes, what can be achieved in manipulating DCs for clinical application, and the future perspective in this area.

### Tumor Microenvironment

Tumor microenvironment is comprised of various types of cells and extracellular components (5). Endothelial cells, fibroblasts, and immune cells make up most TME cell components (6); proteins, glycoproteins, and proteoglycans are functioning as a scaffold (7); cytokines, growth factors, enzymes, and hormones play their regulatory roles in it (8). Tumors cells need high levels of nutrient supply to maintain a high rate of cell proliferation; as a result, it continuously remodels TME to meet the high anabolic demand and energy production rate (9). Changes in TME always make the situation in favor of tumor development (10, 11).

On the one hand, tumor cells secrete blood vessel growth factors which form a new abnormal vessel to maximize nutrients supply (12). Thereinto, the glucose supply is mainly derived from blood and can be maintained through metabolic communication with adjacent tumor cells (13). Tumor cells in the relative hypoxia area usually undergo anaerobic glycolysis and produce a large amount of lactate, which can be discharged to TME by monocarboxylate transporters 4 (MCT4) and transported to the tumor cells with sufficient oxygen supply for consumption by MCT1 to prevent local accumulation of lactate (8). In addition to the increase in the synthesis of fatty acids in tumor cells, fat cells in the TME can also provide fatty acids to tumor cells and accelerate the division and proliferation of tumor cells (14). In addition to the vascular supply, amino acids in TME are derived from soluble proteins in the interstitial fluid. These proteins come from living cells that have been eaten and from apoptotic cells that have been recycled. Tumor cells can absorb them into vesicles by macropinocytosis and restore them to free amino acids by lysosomes (15).

On the other hand, TME can reprogram the metabolism of an immune cell (16), reducing immune activation signals, downregulating antigen recognition and presentation, and making tumor cells escape from host immune surveillance (17), thus causing tumor immune escape. Early invasion of immune cells, such as macrophages, lymphocytes, natural killer (NK) cells, and DCs, is essential for anti-tumor immunity. However, the anti-tumor immune response produced by these cells is inhibited by immunosuppressive cells, such as a bone marrow myeloid-derived suppressor cell (MDSC), a regulatory T cell (Treg), and a type 2 polarized macrophage (M2) (18). As a cell that plays a key role in TME, an MDSC has several mechanisms for immunosuppression (19). On the metabolic level, an MDSC upregulated the expression of arginase 1 (ARG1) and oxide synthase 2 (NOS2) (20). It also increased the uptake of cationic amino acid transporter 2B (CAT2B) and glutamate/cystine antiporter solute carrier family 7 member 11 (SLC7A11), thereby increasing the consumption and intracellular degradation of arginine (Arg) and cystine, exerting an immunosuppressive effect (21). The main metabolic regulation mechanism of Tregs in immunosuppression is the metabolism regulation of tryptophan (Trp) and adenosine. The expression of indoleamine 2, 3-dioxygenase (IDO) in antigen presenting cells was upregulated to induce the depletion of key metabolized amino acids (22, 23). Tumor-associated macrophages are considered as M2, which can induce angiogenesis and increase the nutritional supply of tumor cells through the high expression of vascular endothelial growth factor (VEGF) (24). As a non-immune cell, fibroblasts could be activated and proliferated in TME through the release of cytokines to recruit immune cells and secrete extracellular matrix remodeling factors and then prompt the formation of fibrous matrix to affect vascular development, which leading to tumor area hypoxia and glycolysis (25). Endothelial cells can further utilize the lactate produced by glycolysis for the angiogenesis process. Fibroblasts use lactate to produce hyaluronic acid to achieve tumor invasion (26).

### DCs in Tumor Immunity

Dendritic cells are major antigen-presenting cells in the human body (27), which can process and present antigenic peptides and express them on MHC for antigen-specific T cells to recognize and induce an antigen-specific immune response. Apart from Langerhans cell, major DC cells can be divided into three categories: the conventional DCs (cDCs), plasmacytoid DCs (pDCs), and monocyte-derived DCs (moDCs). cDCs can be further classified into two subtypes: cDC1 and cDC2, according to surface molecules and transcription factors (28). cDCs originate from bone-marrow-derived precursors, which can induce T-cell dependent adaptive immunity (29). pDCs also belong to bone marrow-derived DCs (BMDCs), which undertake the role of type I interferons (IFN-I) producer. moDCs are always differentiated from monocytes; it is a reaction of inflammation and exists in specific tissues in a steady-state (30, 31). Under steady circumstances, DCs are immature antigen-presenting cells (APCs), lack the expression of costimulatory molecules, seldom secrete cytokines, and reside at peripheral tissues (32). However, immature DCs have a potent ability to capture antigens. Activated DCs can express more MHC II, C-C chemokine receptor type 7 (CCR7) (33), and costimulatory molecules and are also able to secrete a variety of cytokines. At the same time, activated DCs downregulate its antigen capturing capacity and gain the ability of migration to draining lymph nodes (dLNs) (34). The marks for DC maturation include the expression of MHC-peptide complexes on the cell surface, the enhancement of costimulatory molecules, the secretion of cytokines, and the ability to activate corresponding T cells (35). DCs can be activated through pattern recognition receptors (PRRs) binding with pathogen-associated molecular pattern (PAMP) molecules, or damage-associated molecular patterns (DAMP) molecules. PAMPs, such as lipopolysaccharides (LPS), endotoxins, bacterial flagellin, lipoteichoic acid, and nucleic acid, can be recognized by toll-like receptor (TLR), which are typical PRR. Endogenous tissue damage fragments, such as tumor cell DNA, belong to the DAMP and can also be identified by PRR. Tumor cells have their specific antigens: cancer-germ line genes antigens, rearrangement gene production, and antigens from a virus (36). Besides, inflammatory cytokines and some ligands can also activate DCs (37). DCs maintain the homeostasis of the body by activating CD8^+^ T cells, keep cancer cells under control, and rely on the presence of the DAMP, their metabolic status, and the activation status, which are critical to anti-tumor immunity *in vivo*.

### Activation of DCs in TME

**Figure 1:**
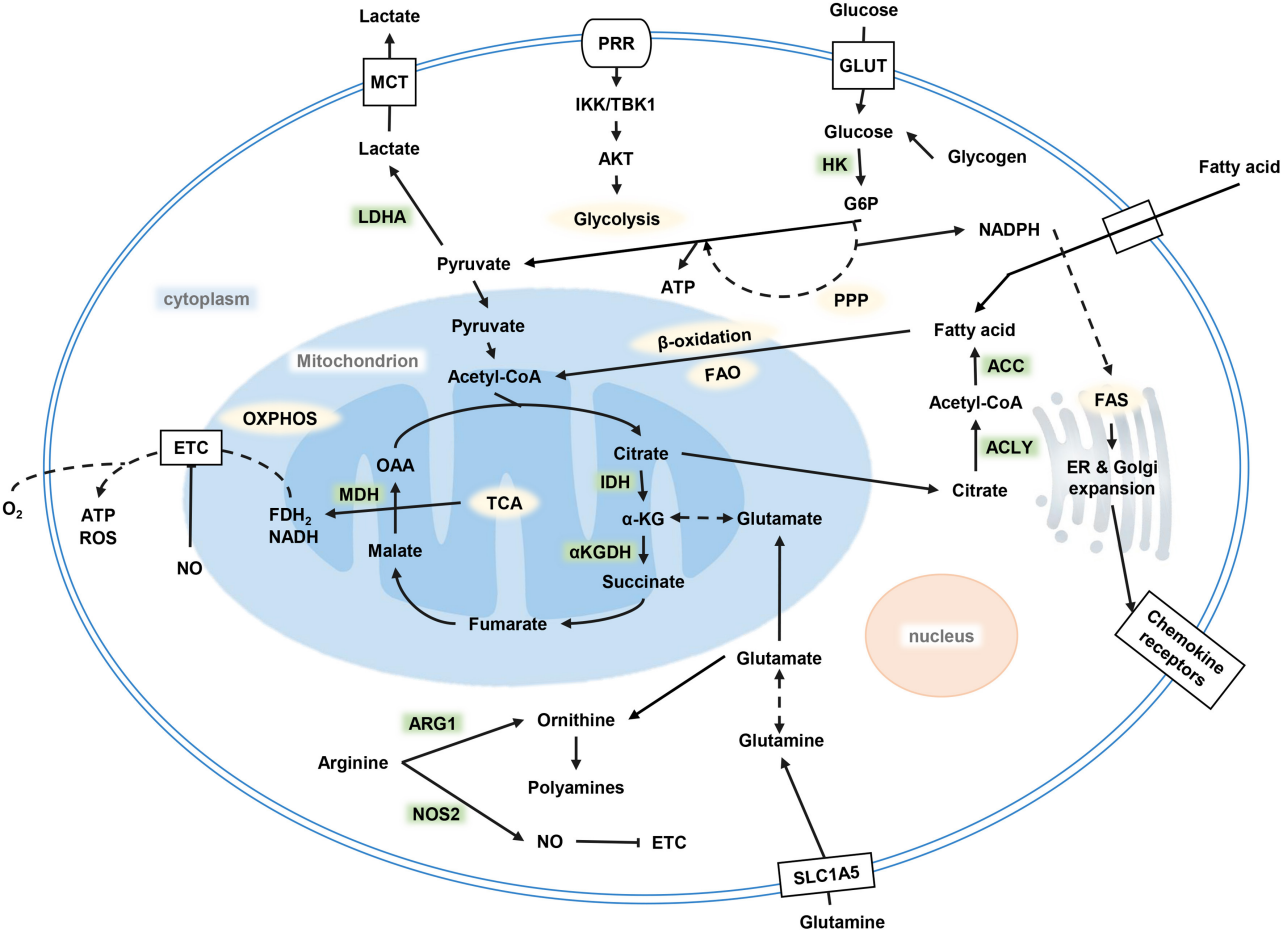
Metabolism of DCs. Glycolysis occurs after DC activation, which is powered by glucose and glycogen to produce ATP and citrate. Citrate provides the raw material for FAS, and FAS provides the necessary conditions for ER and Golgi expansion after DC activation to complete a series of functional activities. OXPHOS decreased while glycolysis increased. DC, dendritic cell; FAS, fatty acid synthesis; ER, endoplasmic reticulum; OXPHOS, oxidative phosphorylation.

After TLRs recognized stimuli to activate DCs, a series of metabolic changes happened in DCs to promote maturation, embodied DC's function of migration and activation of the corresponding effector T cells. Immediately after the TLR ligand response, DCs upregulate glucose uptake and lactate production mediated by the phosphoinositide 3-kinase (PI3K) / serine/threonine kinase (AKT) pathway, and the TANK binding kinase 1 (TBK1)-IκB kinase ε (IKKε) pathway. Glycolysis produces NADPH by the pentose phosphate pathway (PPP) and citrate by the tricarboxylic acid cycle (TCA) (Figure 1). On electron transport chain (ETC), reactive oxygen species (ROS) is produced during electron transfer when ADP is converted to ATP. Physiological doses of ROS help protect antigens from degradation in DCs by the endocytosis chamber, mainly mediated by the inhibition of acid lysosomal proteases (38). Citrate, together with NADPH, are exported to the cytoplasm to provide fuel for fatty acid synthesis (FAS). Citrate converting into acetyl-CoA and incorporating into FAS are necessary steps for endoplasmic reticulum (ER) and Golgi expansion. ER expansion is common in DCs when it is activated and has demand for protein secretions, which in turn lead to unfolded protein accumulation. This process results in ER stress; the unfolded protein response (UPR) ensues the handling of this situation in cells, followed by accelerated FAS and folding-related protein synthesis (39). The uniqueness of citrate utilization in DCs is considered as being a key event in supporting DC activation, maturation, and its specialization in biological functions (40).

## Glucose Metabolism

### Glucose Metabolism of DCs

The significant increase in glucose consumption and lactate production after the activation of DCs by TLR agonists results from a rapid increase of glycolytic flux within minutes of TLR agonists, stimulating all cDCs (41). However, the stimulation received by TLR can be strong or weak, but early glycolytic activation of DC always occurs. The difference is the activated DCs showing different biological energy distribution with different stimulus intensity. DC activation by strong TLR agonist (such as LPS) shows an increase in glycolysis and the elimination of ATP-coupled mitochondrial respiration and a corresponding decrease in oxidative phosphorylation (OXPHOS). However, DCs activated by weak activators, such as house dust mite (HDM), also rapidly increase glycolysis in the early stage after stimulation but lack long-term glycolytic reprogramming, which is needed to depend on hypoxia-inducible factor 1α (HIF-1α) to retain mitochondrial oxidative metabolism. DCs that received stronger stimulation show higher levels of glucose metabolism than those who received weak stimulation.

There are two stages of metabolic reprogramming in DCs after its activation. Different signaling cascades are responsible for the events. First, TLR activation initiates acute glycolysis in DCs within minutes to support the high biosynthetic requirements associated with early DC maturation in the coming hours. Then, nitric oxide (NO)-producing DCs proceed with long-term glycolysis. The acute glycolytic reprogramming of DC occurs within few minutes of TLR stimulation, independent of mammalian target of rapamycin (mTOR) but regulated by the PI3K/TBK1/IKKε/AKT signal axis. With the signal transduction, hexokinase 2 (HK2) rapidly translocates from the cytoplasm to mitochondria to support rapid glucose catabolism, which is required for DC maturation. After glucose decomposing, it enters PPP to produce lactate and synthesize into citrate, which is used for subsequent synthesis of fatty acids and cytokines.

In NOS2 expressed DCs, mammalian target of rapamycin complex 1 (mTORC1) /HIF-1α can induce NO production and impair the activities of the ETC in mitochondria through NOS2, promoting aerobic glycolysis. However, most DCs are NOS2-deficient and therefore rely on mechanisms, such as IFN-I, HIF-1α, TBK1, and IKKε, to maintain glycolysis. Meanwhile, exogenous NO also promotes the stabilization of HIF-1α and plays a certain role in maintaining glycolysis. Aerobic glycolysis occurs mainly because the immune system needs to respond quickly, although glycolysis produces ATP less efficiently than OXPHOS does, but this pathway is faster.

Extracellular glucose input and intracellular glycogen decomposition make up the consumed glucose (42). During the activation of DCs, except extracellular glucose to support DC activation, intracellular glycogen storage also plays an important role. Glycogen is a large branched-chain polymer of glucose found in the liver, muscle, and nerve tissues. The expression of glucose transporter 1 (GLUT1) is thought to support DC activation-related glycolysis. However, in DCs, GLUT1 upregulation occurs hours after TLR stimulation, but TLR-mediated glycolytic reprogramming occurs within minutes of activation. Thus, the glucose that supports the earliest events in DC activation is thought to come from intracellular glycogen (43). DCs possess intracellular glycogen storage and have the mechanism to catabolize intracellular glycogen, which can support its activation and effector functions. Glycogen-derived carbon preferentially supports the synthesis of citrate. Inhibition of glycogen catabolism impairs the function of DCs (44).

*In vitro*, glucose is critical for DCs migration to C-C motif chemokine ligand 21 (CCL21). Blocking glycolysis serves to destroy the optimal migration of DCs to dLNs. The inhibition of glycolysis leads to the damage of CCR7 oligomerization and reduces the ability of movement of DC significantly. At the same time, DCs lose the dynamic and elongated phenotype and present a circular shape, considered as a movement that defected DCs. This also suggests that glycolysis may play a key role in cytoskeletal remodeling (45).

### Glucose Metabolic Changes of DCs in TME

Activated DCs rely on glycolysis and PPP to maintain their energy production and membrane integrity, provide elements for the generation of an inflammatory mediator, and sustain their ability to migrate (17). Inhibition of glycolysis impairs the functions of DCs, including antigen presentation, cytokine production, and T-cell stimulation. The upregulation of MHC II expression on the surface of DCs needs molecule redistribution of endocytic compartments through lysosome tubulation, which needs energy support. In addition, the process of peptides loading onto MHC II needs lysosomal compartment acidification. The process needs ATP coming from glycolysis (34). TLR-induced costimulatory molecule expression and cytokine secretion also need energy from glycolysis, CD40, CD86, the costimulatory molecules and interleukin (IL)-12, the key polarized cytokines of T helper (Th) 1 are important factors in DC function. Meanwhile, some other studies showed that inhibiting glycolysis 8 h after the initial activation of DCs did not result in the functional inhibition of DCs but enhances the ability of DCs to induce T cells, which may be due to DCs having reached dLNs by the time the DCs were studied in Kedia-Mehta and Finlay (46).

After DC activation, many effective T cells will be activated. When multiple T cells interact with DCs, T cells will consume a large number of nutrients, thus affecting the utilization of glucose by DC. The competitive uptake of glucose by activated T cells can disrupt the glucose supply of DC, inactivate the mTORC1/HIF-1α/NOS2 glucose-sensing signaling pathway, and promote the output of pro-inflammatory DC to enhance the response of T cells.

As an important metabolite of glycolysis, lactate has important influence on the activation and function of DC in many aspects. The lactate in the TME can promote tumor growth through G-protein-coupled receptor (GPR81), a g-protein-coupled lactate receptor. Lactate can activate GPR81 on DCs of mice to inhibit MHC II presentation on the DC surface. It also has an association between GPR81 activation on DCs and the reduction of cyclic adenosine monophosphate (cAMP), IL-6, and IL-12 (47). Lactate can also attenuate IFN induction in pDC through GPR81 receptor or directly introduce lactate into the cytoplasm through monocarboxylic acid transporter in pDC, affecting cell metabolism required for pDC activation. Another mechanism is to induce Treg production by promoting the tryptophan metabolism of pDC and generating kynurenine (Kyn) (48). The absence of GPR81 can inhibit tumor growth and promote tumor immunotherapy (49). At the same time, the lactate in TME can inhibit TLR3 and its downstream IFN-I and the stimulator of interferon genes (STING), which accelerates the degradation of antigen, affects the cross-presentation ability of DCs, and thus fails to initiate the anti-tumor response (50). Lactate dehydrogenase (LDH) is a metabolic enzyme that catalyzes the conversion between lactate and pyruvate. Tumor cells activate HIF-1α and c-Myc due to hypoxia, and these transcription factors bind to the promoter of LDHA, upregulating the expression of LDHA and converting more pyruvate to lactate (51). The accumulation of lactate in TME inhibits the activation and antigen expression of DCs. Therefore, the high expression of LDH is also considered as a biomarker with poor prognosis (52).

Glycogen is not only an important source of DC-activated glucose but also an important substance for tumor cell growth. In a study of pancreatic cancer, tumor cells deficient in HIF-1α had glycogen accumulation and inflammatory cytokines secretion, which recruited cDC into tumor stroma. cDCs secrete cytokines for promoting tumor angiogenesis (53). The accumulation of glycogen in tumor cells makes cDCs have completely opposite functions as usual (54). Metabolism of tumor cells, such as lactate production by glycolysis and glutamine catabolism, formed an acidic TME. The acidification of TME obstructs the ability of DCs for antigen uptake and the stability of antigen-MHC-I complex. The negative or positive effect of pH for antigen uptake depends on the receptors combined with antigen, and the antigen-MHC-I complex preferred a neutral environment (55). Mannan receptor (MR), as a receptor expressed in a variety of APCs included DCs, is also affected by pH. The low pH of TME reduces the ability of MR to bind antigens. At the same time, however, low pH in TME inhibited glycolysis and lactate production, increased mitochondrial respiration, and downregulated the activity of mTORC1. In this environment, monocytes are more likely to differentiate into moDCs (56).

There is often more ROS storage in TME due to the high glucose metabolism of tumor cells. As a by-product of ETC, ROS has different effects on tumor and immune cells at different concentrations (57). At a low concentration, it can promote cell proliferation, differentiation, migration, and angiogenesis. With the increase of ROS concentration, the stress response of cells will be activated, causing inflammation, fibrogenesis, tumor growth, invasion, and even cell death (58). High ROS levels in TME can lead to DNA, protein, and lipid damage, and tumor cells have the ability to remove more ROS due to their antioxidant defense function (59). A high level of ROS often leads to abnormal activation of DC, possibly because of the oxidation of high mobility group protein B1 (HMGB1), an important molecule for inducing DC maturation, by ROS, leading to T-cell dysfunction (60).

## Lipid Metabolism

### Lipid Metabolism of DCs

Lipids, including fatty acids, triglycerides, cholesterol, phospholipids, and sphingolipids, are precursors of many molecules that have important biological roles (61). Lipids which are widely distributed in organelles, such as cholesterol and fatty acids, are the main components of cell membranes; lipids can provide energy in the absence of nutrients (62) or synthesize more complex fat-containing substances (63) and can serve as second messengers in cells to transmit signals (64). Activation of DCs is associated with an increased ability to capture and present antigens and an increase in the synthesis of cell surface proteins or secretory proteins. This process is regulated by the expansion of fatty acid synthesis depending on the ER and Golgi body network in BMDCs stimulated by granulocyte-macrophage colony-stimulating factor (GM-CSF). During DC activation, ER and Golgi bodies always expand to support the synthesis of fatty acids after TLR-driven glycolysis. There is an increased demand for biosynthesis in activated DCs. Intermediates produced by glycolysis and mitochondrial metabolism, such as acetyl-CoA, are nutrient sources for *de novo* synthesis of fatty acids and are essential for DC activation (37). When DCs are stimulated to mature *in vitro*, the accumulation of fat and glycogen occurs in DCs cells. The promotion of fatty acid synthesis in response to TLR stimulation also leads to an increase in lipid storage in lipid droplets.

Fatty acid metabolism is involved in DC development, maturation, and function. With its integration with mitochondrial function, the FAS affects DC derivation, which can not only block moDC formation from human PBMC but also prevent the generation of DCs in primary and secondary lymphoid organs. At the meantime, FAS decreases MHC II and increases CD40 expression on the DC surface. As a key transcription factor regulating lipid metabolism, peroxisome proliferator-activated receptor (PPAR) was found to be significantly upregulated *in vitro* in moDCs induced by the GM-CSF and IL-4 (65). Saturated and polyunsaturated fatty acids are agonists of TLR4, which can promote the expression of pro-inflammatory transcription factors. However, in mature DCs, high-density lipoprotein and low-density lipoprotein will damage the TLR4 signaling (66). Arachidonic acid and eicosapentaenoic acid can affect moDCs differentiation, cytokine production, and T-cell stimulation. Studies have shown that lauric acid can stimulate LPS-induced DC maturation and facilitate T-cell activation, while docosahexaenoic acid (DHA) plays an opposite role and can inhibit the same DC maturation. Besides, DC shows a tolerogenic phenotype after vitamin D3 treatment.

Several studies have shown that fatty acid metabolism is also important for tolerogenic DCs. The oxidative activity of fatty acids in tolerogenic DCs is higher than that in mature DCs, and the decrease of fatty acid production leads to the decrease of immunogenicity in DCs. Mature DCs tend to choose the glycolytic metabolic pathway and preferentially use glucose as a carbon source. In contrast, tolerogenic DCs were more prone to have OXPHOS and fatty acid oxidation (FAO) pathways. This metabolic reprogramming of DCs results in a different status in DC cell function (67). While tolerogenic DCs shift cell metabolism to OXPHOS and FAO, this highly decomposable energy spectrum may be associated with the large amount of energy required for inhibitory activities and protein degradation (68).

### Lipid Metabolic Changes of DCs in TME

Abnormal accumulation of lipids in DCs is one of the main mechanisms of DCs dysfunction. Lipid accumulation in DC can reduce antigen handling capacity, downregulate co-stimulating molecule CD86, and overexpress tolerogenic cytokine IL-10 (69). The mechanism for lipid accumulation can be increased by fatty acid synthesis or lipid uptake from plasma (67). In ovarian cancer, the expression of fatty acids synthase (FASN), the key enzyme of *de novo* lipogenesis, was found increased. The upregulated FASN leads to an increase of fatty acids synthesis in ovarian cancer cells, and the high concentration of fatty acids in TME results in fatty acids accumulation in DCs, thus affecting its function. Targeting FASN upregulation of the tumor-promoting pathway can enhance anti-tumor immunity (70). A study in hepatocellular carcinoma (HCC) found the upregulation of FAS-related genes in most HCC tissues. At the same time, DCs can express scavenging receptors to promote the accumulation of lipids in cells, resulting in a reduced expression of costimulatory molecules and cytokines, reducing its ability to activate T cells. This phenomenon mainly occurs in cDCs but not in pDCs (71). The intratumoral infiltration of pDCs is considered as one factor associated with poor prognosis, because of their ability to induce Tregs and promote IL-7 secretion (72). Cetyl-CoA carboxylase inhibitor can normalize lipid abundance in DCs and restore DC function (73).

Studies have shown that the accumulation of oxidized lipids, especially triacylglycerol (TAG), can cause DC dysfunction and shorten its life span. The increased TAG level in DCs of lymphoma mouse or patients with lymphoma is mainly realized by regulating the expression levels of scavenger receptor A, lipoprotein lipase, and fatty acid-binding protein 4, and promoting the uptake of TAG in BMDCs and moDCs (74). Consistent with these findings, lipid droplet accumulation in ovarian cancer is also to be responsible for the failure of DCs to induce an anti-tumor T-cell response, and the dysfunctions of DCs in radiation-induced thymic lymphoma and mesothelioma are also because of lipid accumulation (74). In lung cancer, the amount of DCs in the peripheral blood of a patient at the initial treatment period is significantly less than that in the healthy control group. The number of moDCs and pDCs is also significantly reduced in stage III and IV patients. In patients with stage IV lung cancer, the lipid accumulation in DCs is significantly higher than that in the control group, with the highest accumulation intensity in moDCs. The accumulated lipids in the cell are identified as TAG (75). moDCs derive from peripheral mononuclear cells, differentiated under the action of GM-CSF and IL-4, and preferentially induce naive T cells into mature Th1 cells (76). When immature moDCs were exposed to mesothelioma cells, their lipid levels were significantly higher than that of the control group and inparallel with tumor progression.

Lipids accumulated in DCs also result in antigen cross-expression impairment, which is mainly caused by a defective transportation of peptide-MHC (pMHC) class I complexes to the cell surface. In a tumor, DCs usually accumulate lipid body (LB) inside, some of them are electrophilic oxidatively truncated (ox-tr) lipids. This ox-tr-LB can covalently bind with heat shock protein 70, which is not found in control group DCs (77). This interaction prevents pMHC transfer to the cell surface, which then causes an accumulation of pMHC in the late endosome/lysosome. Therefore, tumor-related DCs cannot stimulate sufficient CD8^+^T cell response (77). In the early days of DC maturity, tumor cells can secrete a-fetoproteina (AFP) to inhibit FAS and mitochondrial metabolism of DC, which is mediated by AFP downregulating the expression of sterol regulatory element-binding protein-1 (SREBP-1) and PPAR-γ coactivator-1α (PGC1-α), the DC metabolism-regulating molecules. These metabolic changes occur as early as 24 h after AFP exposure (78).

Accumulation of fatty acids strengthens FAO, which leads to tumor immune tolerance (41). In melanoma, DC cells through the Wnt5a-β-catenin-PPAR-γ signaling pathway can upregulate the expression of carnitine palmitoyltransferase-1a (CPT1A) fatty acid transporter protein, drive the FAO process, promote the development of Tregs, inhibit the activation of effector T cells, and establish immune privilege sites (79). Blocking this pathway can enhance anti-melanoma immunity, enhance anti-programmed death-1 (PD-1) antibody immunotherapy efficiency, and inhibit disease progression (79). Tumor cells can secrete fat-containing exosomes as a fatty acid carrier and activate PPARα of DCs to induce the synthesis of lipid droplets in DCs and enhance FAO, inducing the transformation of DC metabolism from glycolysis to OXPHOS, leading to the dysfunction of DC. As a key molecule of metabolic regulation, PPARα can be used as a target of immunotherapy and has a great potential in anti-tumor therapy (80).

Recent studies have highlighted the complexity of lipid metabolism in regulating DC function. In normal conditions, inositol-requiring enzyme-1α (IRE1α)–X-box-binding protein 1 (XBP1) signal is essential for DC function (81). However, DCs in tumors can accumulate oxidized lipids, inhibit T cell function, and promote the progress of the tumor. The specific mechanism is considered through activating the ER stress response *via* IRE1 and then activate XBP1, which promotes the synthesis and accumulation of fatty acids and triacylglyceride and thereby decreases DC immunogenicity (82). Therefore, removing XBP1 from DCs can enhance the immunogenicity of DCs and initiate a protective immune response against the tumor. Removal of vitamin E by ROS also improved ER stress response in DCs (82). Vitamin D3-treated DCs can induce the expression of immunoglobulins like transcript 3 (ILT3), which leads to the amplification of ILT3-dependent CD4^+^Foxp3^+^Tregs and the immunosuppression (83). It also has been reported that 1,25-(OH)_2_D3, the active form of vitamin D3, can induce a tolerogenic phenotype of DC by activating the PI3K/AKT/mTOR dependent glycolysis in moDCs for metabolic reprogramming (84). Binding of vitamin D3 to vitamin D receptor significantly increases the binding affinity of nuclear factor-κB (NF-κB), inhibits the activation and transcription activation of NF-κB, and induces tolerogenic DCs formation. DC treated with BAY 11-7082 (NF-κB inhibitor) shows a low expression of MHC II and CD40 molecules, and the DC treated with injection BAY 11-7082 *in vivo* induce the production of CD4^+^ Tregs (68). Lipid metabolism of DC can also be affected by the state of TME acidosis. Malignant mesothelioma (MM) cells produce a large amount of lactate due to aerobic glycolysis, which result in acidosis and transforming growth factor (TGF)-β secretion. A large amount of lipid droplets formed in DCs, leading to a decreased ability of DCs to migrate to lymph nodes or activate T cells (85).

## Amino Acid Metabolism

### Amino Acid Metabolism in DCs

Under normal circumstances, the synthesis of amino acids in DC is essential for the function of DC. After TLR receives a stimulation, an increasing mitochondrial content and intracellular glutamine (Gln) in an autophagy-dependent manner can change the expression of genes related to glutamine metabolism in pDCs (86). Glutamine decomposes for fueling glycolysis and transforms into α-ketoglutarate (α-KG) to support the TCA through glutaminolysis in normal conditions. It also is one of the essential components in producing uridine diphosphate n-acetylglucosamine (UDP-GlcNAc), which is a very important element for maintaining the expression of transcription factor c-Myc. mTORC1 can induce the expression of c-Myc, which also depends on the availability of amino acids and regulating glutaminolysis. c-Myc protein has a short half-life span in lymphocytes and can continuously express only in cells with high amino acid uptake rate and high protein synthesis rate (87).

### Amino Acid Metabolic Changes of DCs in TME

**Figure 2:**
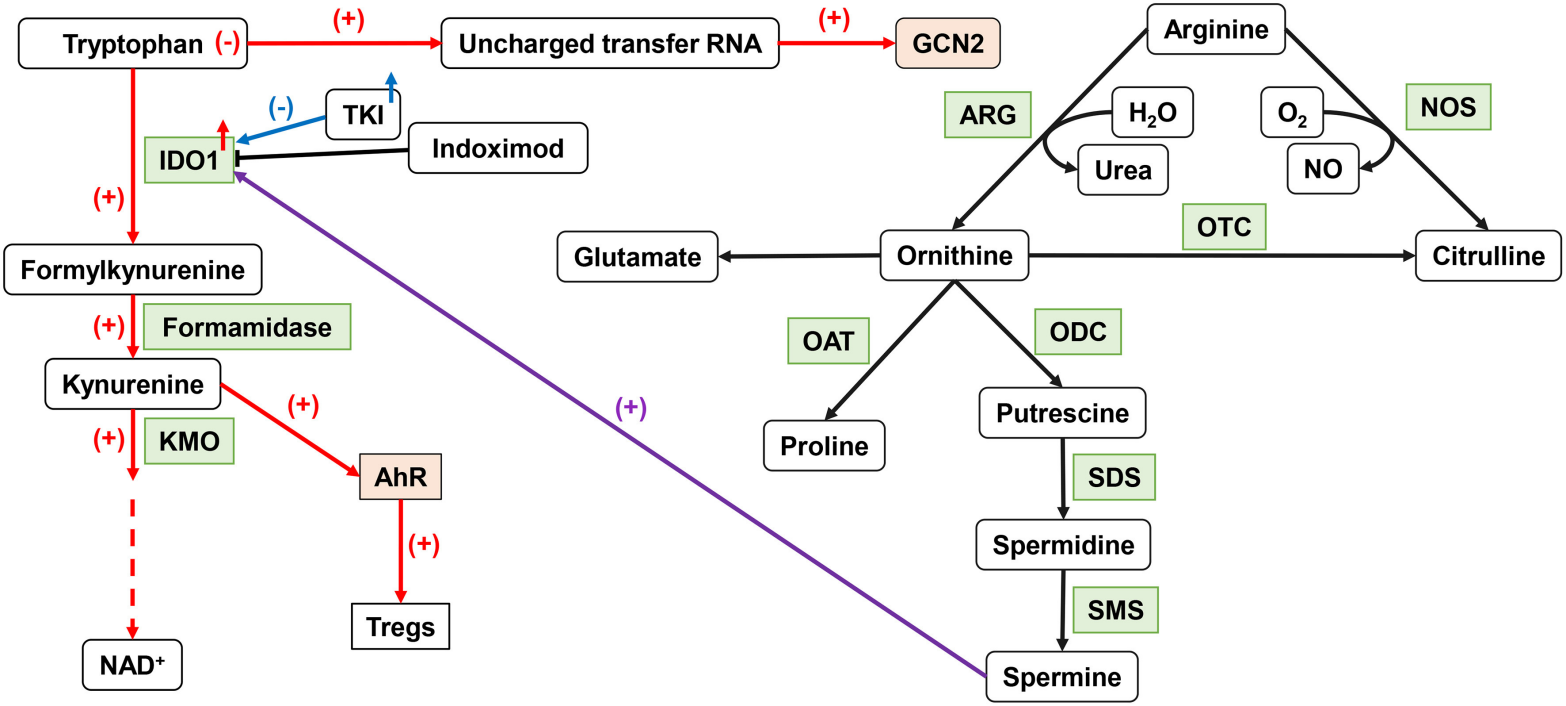
Important amino acid metabolism in DCs. IDO1 can affect DC function by tryptophan metabolism. The upregulation of IDO activity leads to the production of Tregs. ARG1 catalyzes arginine to produce ornithine to produce spermine, which can affect IDO1 function through an intermediate pathway. NO, produced by NOS metabolism, inhibits the function of ETC. IDO1, indoleamine 2,3-dioxygenase 1; ARG, arginase; NO, nitric oxide; ETC, electron transport chain.

Trp is an essential amino acid in the human body, involved in the *de novo* synthesis of nicotinamide adenine dinucleotide (NAD^+^) (88). Its metabolic status can affect the anti-tumor immune function (Figure 2). IDO, a member of the heme dioxygenase family, is a rate-limiting enzyme for the catabolism of Trp, which can be expressed and secreted by both tumor cells and immune cells in the TME (89). After IDO decomposed Trp and product Kyn, the Trp deprivation happened locally, and the metabolite affects immune microenvironment. Kyn can be used as an aryl hydrocarbon receptor (AhR) agonist to induce the expression of AhR in DCs (90). The downstream Trp metabolite of Kyn, 3-hydroxyanthranilic acid (3-HAA), can directly target nuclear coactivator 7 (NCOA7) to increase the transcriptional activity of AhR in cDCs and induce the generation of Treg (91), which also induces other DCs to produce IDO1 through its interaction with cytotoxic T-lymphocyte antigen 4 (CTLA4) (92). At the same time, AhR can in turn upregulate the expression of IDO, thus exacerbating the immunosuppressive effect (46). Kyn itself can also act as a signaling molecule to block the anti-tumor immune response (93). The absence of Trp increases uncharged transfer RNA (tRNA), leading to a comprehensive stress response mediated by general control non-derepressible 2 (GCN2). GCN2 is a direct sensor for a low level of cellular amino acids and is activated to stimulate free radical reprogramming of cell functions, leading to immune cell cycle arrest and autophagy when the acquisition of cell amino acids is limited (94). Trp depletion also can directly activate GCN2, which promotes Treg differentiation and also inhibits T-cell function (95). However, tumor cells can upregulate the expression of amino acid transporters and regulate the sensitivity of amino acid sensors of itself such as GCN2 and mTOR signals to respond to amino acid reduction.

As a semi-essential amino acid, Arg is a major precursor for the synthesis of cancer-related compounds, such as NOS, NO, and polyamines (96). Both NOS and ARG1 catalyzed Arg decomposition. ARG1 breaks down Arg to produce urea and L-ornithine (Orn) for further protein synthesis. NOS can use Arg to produce NO and L-citrulline. TGF-β can induce the co-expression of IDO1 and ARG1 in DCs. There is also an intermediary pathway between IDO1 and ARG1: spermine, the downstream product of ARG1, can induce IDO1 in DC. Phosphorylation of IDO1 in DCs and the subsequent activation of IDO1 signals strictly depend on the prior expression of ARG1 and the production of ARG1-dependent polyamines. Polyamines can regulate DC differentiation to IDO1-dependent immunosuppressive phenotypes by activating steroid receptor coactivator (SRC) 1 kinase, which includes IDO1 phosphorylation activity (97). The expression of IDO, ARG1, or NOS2 will lead to a decrease of Trp and Arg levels and affect the nutrition-related signaling pathway. Decreased amino acid levels also affect the functional status of immune cells.

## Metabolic Sensor of DCs in TME

### mTOR Signaling

Cell energy catabolism is characterized by increased energy decomposition and over energy consumption, which is controlled by mTOR. There are two stages of metabolism transformation of DCs from OXPHOS to glycolysis: early glycolysis is NOS2 independent and mediated by TBK1-IKKε-AKT pathway and long-term glycolysis is raised by PI3K-AKT-mTOR signal (98). mTOR forms two complexes, the mTORC1 and mammalian target of rapamycin complex 2 (mTORC2), which regulated DC metabolism in several aspects (99).

**Figure 3:**
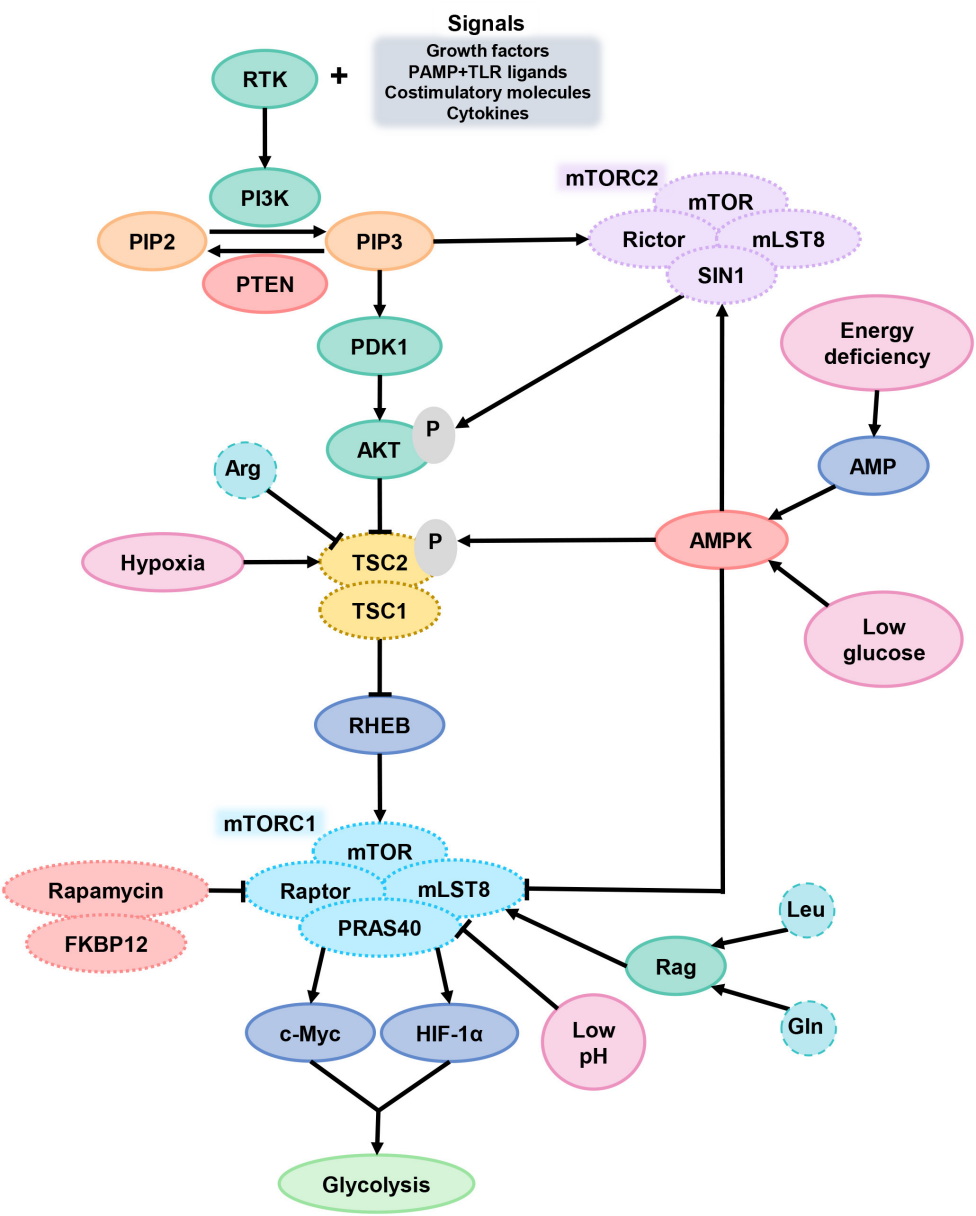
mTOR signaling in DCs. After DCs received the extracellular signals and activated the signal intermediate PI3K, PIP2 transformed into PIP3 and then activated AKT. AKT activates mTORC1 through TSC2 inactivation. When cells are in TME of hypoxia, energy deficiency, glucose deprivation or low pH, the function of mTORC1 is inhibited. PTEN and AMPK are the two main ways of inhibiting mTORC1. PI3K, phosphoinositide 3-kinase; PIP2, phosphatidylinositol-4, 5-bisphosphate; PIP3, phosphatidylinositol-3, 4, 5-trisphosphate; PTEN, phosphatase and tensin homolog; AKT, serine/threonine kinase; TSC2, tuberous sclerosis complex protein 2; mTORC1, mammalian target of rapamycin complex 1.

In general, mTORC1 acts as a key component of metabolic regulation that can be activated by growth factors, cytokines, and chemokines, which bind to cell surface molecular such as receptor tyrosine kinases (RTKs) and activate signal intermediate like PI3K (Figure 3). Then, phosphatidylinositol-4, 5-bisphosphate (PIP2) convert into phosphatidylinositol-3, 4, 5-trisphosphate (PIP3) and recruit pleckstrin homologous (PH) domain proteins (such as AKT, mTORC2, and pyruvate dehydrogenase kinase isozyme 1), resulting in AKT activation. Finally, AKT activates mTORC1 and promotes protein synthesis in cells (100). Phosphorylation of AKT inactivates the tuberous sclerosis complex protein (TSC) 2, which inhibits mTOR by forming complexes with TSC1. mTOR can be activated by TSC2 inhibition (101). In general, two major ways to inhibit mTOR is phosphatase and tensin homolog (PTEN) or AMP-activated protein kinase (AMPK) activation. Both PTEN makes PIP3 dephosphorylation and AMPK causes TSC2 phosphorylation, which can lead to mTORC1 inactivation (102).

mTORC1 can promote glycolysis by improving the activity of transcription factors c-Myc and HIF-1α and is a central factor in coordinating the metabolic changes and immune response of DCs (103). The mTORC1 signaling pathway is sensitive to amino acid sufficiency through Ras-related GTPases (Rag), especially Arg, Gln, and leucine (Leu). Arg can inhibit TSC2 function for mTORC1 excitation. In a physiological situation, the decrease of glucose-6-phosphate level in extracellular fluid will facilitate the combination of mTORC1 and HK2, which caused the mTORC1 inhibition. DC hypoxia provokes DNA damage-induced transcript 4 (DDIT4) expression, which also suppress mTOR function *via* TSC2 (104).

mTOR is a double-edged sword of DC metabolism regulation. On the one hand, it is dispensable for DC polarization and development (105). After PRR signal activates DC, mTOR work to support DC energy anabolism *via* inducting the synthesis of nucleic acids, proteins, and lipids (106). For sustaining glycolysis, mTOR inhibits OXPHOS by regulating the NOS expression to product NO (107). On the other hand, the effect on DC will change under an abnormal conditions. Constitutive activation of mTORC1 can reduce the expression of MHC II by downregulating complex trans-activator (CIITA), the key factor for MHC II expression. At the same time, mTORC1 abnormal activation raised by TSC1 deletion will upregulate acetyl coenzyme A carboxase 1 (ACC1) instead and impairs DC development, further accelerating DC maturation and making cell apoptosis. mTOR or Raptor knockout can reverse this effect on DCs (108). In TME, low pH inhibits the activity of mTORC1 and also inhibits glycolysis.

The main reason for the functional difference of mTOR in DC is the spatiotemporal dependency. From the perspective of time, after the activation of DCs and generation pro-inflammatory signals, inhibition of mTOR has anti-inflammatory effects. With the migration of DC to lymph nodes and activation of T cells, the antigen presentation capacity of DC is turned off and the expression of programmed death-ligand 1 (PD-L1) and IL-10 is replaced. At this stage, the lifespan of DC is shortened due to energy consumption and metabolic transformation, and inhibition of mTOR at this phase can enhance the antigen presentation ability of DC. In terms of space, the concentration of glucose, oxygen, lactate, and amino acids around different tissues is divergent and can be sensed by mTOR. T cells activated by DCs, will compete glucose with DCs, thus caused mTOR downregulated in DCs. At this stage, drug inhibition of mTOR accentuates the signal supporting T cell activation (109).

### Hypoxia and HIF

The expression of HIF-1α is an important elements for immune response process in DCs. Under hypoxia circumstance, HIF-1α can promote the expression of glycolysis gene for more energy supply, such as GLUT1 and LDH (110). HIF can also regulate the generation of ATP and ROS (111). After TLR4 activates moDCs, glycolysis in DCs is sustained by increasing the activity of HK2 through p38-mitogen-activated protein kinases (MAPK)-dependent HIF-1α accumulation, which promotes the initial rate-limiting step of glycolysis (112). In moDCs, HIF-1α also enhances TNF-α release by acting on MAPK kinase kinase 8 (MAP3K8), an upstream molecule of p38-MAPK (113). Combined hypoxia with HIF-1α also increases the production of costimulatory molecules and pro-inflammatory cytokines, and upregulates the expression of PRR, complement receptor components, and immunomodulatory receptors (114).

In solid tumors, vascular structure deficiency causes severe hypoxia of TME, leading to metabolism changes and dysfunction of DCs, such as excessive adenosine accumulation and IDO high expression, making DCs lose its function (115). After tumor radiotherapy, a large amount of ATP was released in TME, and the expression of adenosine generating enzyme CD73 was increased, which also inhibited the function of DCs. Anti-tumor ability of DCs can be restored after blocking CD73 (116). Hypoxia of TME will affect the differentiation, antigen uptake, and migration ability of DCs (117, 118). Long-term hypoxia inhibiting the migration of DCs through upregulate the expression of HIF-1α, while short-term hypoxia has been reported to enhance the migration ability of moDCs (119). Another study further confirmed this conclusion, an lncRNA lnc-Dpf3 was found to inhibit HIF-1 function by directly binding to HIF-1, and further impair glycolysis and inhibit CCR7-mediated DC migration (120). Hypoxic TME upregulated the HIF-1α expression and activate the death program of immature DCs, leading to early apoptosis. On the contrary, after DCs was stimulated by LPS *in vitro* can inhibit apoptosis through HIF-1α (121), but it has also been reported that DCs can be converted to autophagy under such similar circumstances (122).

## DCs and Anti-Tumor Immunotherapy

### DCs as a Target of Immunotherapy

In recent years, methods of producing DCs on a large scale from peripheral mononuclear cells have been developed, and an increasing number of DC-based cancer vaccines have been applied in a clinical trial in many cancer types. DCs loaded with tumor-specific antigen can stimulate the immune system of a patient, hence inducing T cells *in vivo* for an antigen-specific anti-tumor immune response (123). With DC cells usually derived from CD34^+^ precursor cells or monocytes, induced by TLR agonists and cytokines (IL-1β, IL-6, TNF-α), prostaglandin E2 (PGE2) are often used to stimulate the maturation of moDCs. Eventually, DCs can express MHC, costimulatory molecules, and cytokines and induce Th1 (124). The success of inducing DC maturation is a decisive factor in the success or failure of DC vaccine treatment.

The main antigen loading methods of DCs include short peptides, long peptides, tumor cell cracking, dendritic and tumor fusion cells, RNA transfection, DNA transfer, and neoantigens. RNA sequencing can also identify mutations in tumors and provide neoantigen in the formation for DC vaccine development (123). Among them, the DC vaccine using tumor cell lysate as the source of tumor antigen has achieved promising results on HCC. The expression of CD83 and CD86 increase significantly after tumor cell lysate loaded into DCs of mice. More tumor antigens and epitopes makes tumor lysate a more effective activator of CD8^+^ cytotoxic T cells and CD4^+^ Th cells. (125).

Although the safety of the DC vaccine has been confirmed, the efficacy is still not satisfactory. Although there are problems such as low antigen delivery efficiency, weak migration ability, and insufficient cytokine release, it still cannot play an effective role in the immunosuppressive TME. Recent research has attempted to address these limitations. The expression of Notch ligand delta-like 1 by mouse OP9 stromal cells can make cDC1 differentiate *in vitro* and have a phenotype closer to cDC1 differentiated under physiological conditions, which were able to express surface markers as well. DCs can also be isolated from the peripheral blood, but the amount of DCs is limited. In particular, cDC1 development is disrupted in cancer patients, such as those with pancreatic and breast cancers, and the available number of DCs is very limited (126).

More recently, new pathways have been explored to activate DCs, one of them is using TLR ligands to induce DC maturation, such as TLR3, 7, and 8 ligands. Although TLRs are present on immune cells, they are also expressed in different tumor cells, such as cells from pancreatic, breast, and ovarian cancers. Activation of TLR in tumor cells creates a TME that damages the immune system, promotes tumor development, and adversely affects the anti-tumor response in TME, and the NF-κB pathway may partly be responsible for the above unfavorable immune reactions (127). As TLR3 on DCs plays an important role in anti-tumor immunity, it is the key factor for the cross-activation of antigen-specific CD8^+^ T cells. TLR3 agonist ARNAX can initiate CD8^+^ T without the production of cytokines, cause tumor regression in mice without systemic inflammation, and can enhance the efficacy of PD-1/PD-L1 blockade (128).

In previous studies, the infiltration of pDCs in tumors is often considered as one factor for poor prognosis for patients with cancer. But in neuroblastoma (NB), TLR-activated pDCs significantly increase the cytotoxic function of dinutuximab-based NK cells through the TNF-related apoptosis-inducing ligand (TRAIL) death-receptor pathway, increase the expression levels of CD69 and TRAIL, and reduce the risk of recurrence in high-risk patients with NB (129).

### Metabolic Changes of DCs Enhance the Anti-tumor Effect

#### Glucose Metabolic Changes of DCs

Mannan receptor can be an important direction for improving DC vaccines as a highly expressed receptor on APC. DCs can target tumor sites more accurately after tumor antigens are modified with mannan, producing more IFN-γ and mediating the production of antigen-specific CD8^+^ T cell (130). It also has a new subgroup of DCs called merocytic DCs (mcDCs) with highly expressed CD11c, and deficiency in CD8a and CD11b has been identified, which used pre-cDCs as precursors and expressing both ZBTB46 and IFN regulatory factor 4 (IRF4). mcDCs have a lower ability to ingest glucose, which resulted in a lower glycolysis dependence than cDC1 and cDC2 and endowed an ability to reverse T cell dysfunction. Therefore, the survival of mcDCs is least affected by glycolytic inhibition and may have stronger adaptability in TME (131).

#### Lipid Metabolic Changes of DCs

As a member of the lipid family, prostaglandins modulated by cyclooxygenase (COX) enzymes are a derivative of arachidonic acid and released by lipid membranes and control the maturity of DCs, cytokine secretion, and T-cell activation. PGE2, an immunomodulator in the inflammatory environment, is one of the most abundant prostaglandins and plays an important role. The complex effect of PGE2 on DCs depends on the site of contact, the type and amount of prostaglandin receptors available, and the maturity of DCs. In peripheral, PGE2 can stimulate the maturation of DCs, upregulate its expression of DC80 and CD86, and cause it migration to lymph nodes. When DCs matured by IFN-α and TNF-α, stimulating further exposure to PGE2 may reduce the expression of CD40 and CD86 and inhibit the presence of DC antigen efficiency (132). Tumor cells can inhibit the function of DC by secreting prostaglandin, downregulating the expression of DC lineage-specific transcription factor ZBTB46, and inducing the extracellular signal-regulated kinase (ERK)/the cyclic AMP response-element binding protein (CREB) signal related to DC differentiation to increase the synthesis of IL-10. Since COX-2 is often absent in healthy tissues, the use of the COX-2 inhibitor NS-398 to reduce prostaglandin synthesis can enhance the anti-tumor potency of DCs by enhancing their immune activity (133). It has been reported that sarcosine treatment of DCs can increase the expression of COX-1 and Pik3cg, which is manifested as the enhanced migration ability of DC, and thus improve the anti-tumor ability, including B16F10 and glioma. This finding could be used to improve the efficacy of DC vaccines (134). There are several new approaches to promoting the maturity of DCs, for example, using picibanil and TLR7/8 ligand CL097 to stimulate the maturation of moDCs while reducing PGE2 can increase the expression of co-stimulating molecules and IL-12p70 without affecting the ability of DC to induce T cells (135).

#### Amino Acid Metabolic Changes of DCs

The IDO pathway inhibitor indoximod can inhibit mTOR activation to reverse the immunosuppressive effect of IDO (136). IDO inhibitor can slow the growth of subcutaneous Lewis lung cancer and B16 melanoma tumor graft, in which the tumor cells do not express IDO, but the formation of tumor induces the continuous increase of IDO production and exert its function on DCs in inflammatory tumor-draining lymph node (TDLN) (137). As an important anti-tumor drug, tyrosine kinase inhibitor (TKI) has been widely used in many kinds of tumors. It can also reduce the phosphorylation level of IDO in DCs, annul the effect of IDO on DCs, and reduce Trp metabolism *via* inhibiting c-Kit. At present, a variety of TKIs have anti-tumor effects, such as dasatinib, which can increase the efficacy of allogenic T cells by changing the DC metabolic status, thus delaying the progress of B16 melanoma in mice (138). After DC was modified with dasatinib, the expression of IDO and Trp metabolism in DCs was downregulated through the c-Kit pathway, and T cell activation was increased. It is suggested that TKI can be used to regulate the metabolism of DC to achieve the purpose of enhancing anti-tumor ability (138). In mature DCs enriched with immunoregulatory molecules, which are sorted by their co-expression of immunoregulatory and maturation genes, the AXL (ARK) receptor tyrosine kinase can induce the upregulation of the PD-L1 (139). ARK is related to tyrosine metabolism, as well as tumor growth, migration, invasion, and epithelial–mesenchymal transition (EMT) (140).

#### Metabolic Sensor of DCs

At present, there are six types of drugs aimed at the PI3K-AKT-mTOR pathway: mTOR inhibitors, active site mTOR inhibitors, pan-class I PI3K inhibitors (PI3Ki), isoform-selective PI3Ki, pan-PI3K-mTOR inhibitors, and AKT inhibitors (141). Effective upregulation of mTOR enables DC to have a stronger ability to initiate T cells. It has been reported that a new type of DCs induced by CD137 ligands was named CD137L-DCs, which have a stronger capacity of T-cell induction. The higher glycolysis efficiency of CD137L-DCs compared with moDCs induced by GM-CSF and IL-4 gives it a stronger anti-tumor ability, and the higher activity of AKT-mTORC1 signal is the primary reason for this glycolysis rate. Inhibiting AKT-mTOR during CD137L-DCs maturation or inhibiting glycolysis after it maturation can make a significant inhibition of CD137L-DCs (98). Studies have shown that using rapamycin to inhibit mTOR will downregulate Cdc42 protein and lead to morphological changes of DC. The decrease of DC surface area and circumference reduces its meeting rate with T cells (142). Therefore, inhibiting mTOR incorrectly reduces the effectiveness of the drug.

### Metabolic Changes of DCs Combined With Immune Checkpoint Blockade

Dendritic cells will overexpress PD-L1 when presenting antigens to T cells to protect themselves from attack. After tumorigenesis, T cells and IFN-γ upregulate the expression of PD-L1 in DCs. T cell immunity induced by DCs was inhibited, providing an opportunity for tumor immune escape and limiting the therapeutic effect of DC vaccines (143). Studies have shown that after tumor stimulation, mitochondrial metabolism in PD-1-deficient myeloid progenitor cells is significantly enhanced, which is related to further differentiation of myeloid progenitor cells. As a result, the metabolic intermediates were increased, especially cholesterol, which promoted the differentiation and antigen presentation of DCs (144). DC vaccine combined with immune checkpoint blockade (ICB) is a promising approach for tumor treatment. A study on the combination of DC vaccine and PD-L1 inhibitor in HCC showed a synergistic effect, which can induce a stronger CD8^+^T cell response, increase the apoptosis of tumor cells, reduce the tumor volume, and prolong the overall survival rate of mice (145).

Pyruvate kinase isoform M2 (PKM2), a key kinase that catalyzes the last rate-limiting step of glycolysis, not only catalyzes the conversion of phosphoenolpyruvate to pyruvate but also affects the expression of PD-L1 on DCs by binding to the hypoxia response elements (HREs) of PD-L1. Conformational change or silencing of PKM2 will reduce the expression of PD-L1 on DCs or tumor cells, but this method combined with ICB has limited therapeutic effect on tumors (146). This may be caused by the killing effect of CD8^+^T cells on DCs after the expression of PD-L1 on DCs was decreased, leading to a decrease in the antigen presentation of tumor infiltrating DC. pDC and MM cells interaction can upregulate the Kyn-3-monooxygenase (KMO) in MM cells and pDCs, and the Trp metabolites in TME are significantly increased. In the co-culture environment of MM and pDC, the combined application of anti-PD-L1 antibody and KMO inhibitor can enhance the activation and anti-tumor ability of T cells and NK cells (147).

CTLA4 can be expressed by tumor cells or immune cells, including DCs, which inhibits T-cell activation by preventing CD80/CD86 on APCs from binding to CD28. Anti-CTLA4 antibody mainly inhibit the function of Tregs by inducing antibody-dependent cell-mediated cytotoxicity (ADCC) or inducing the Treg consumption, directly. Anti-CTLA4 antibody also has a synergistic effect with DC vaccine (148).

## Conclusions

Undoubtedly, if the metabolism of DCs in TME can be controlled at an ideal status, the function of DCs in the tumor can be regulated. As a crucial APC, DCs are responsible for recognizing and presenting tumor-related antigens, expressing co-stimulating molecules, secreting cytokines, and activating T cells for anti-tumor immunity. It has great potential to be a powerful tool at the starting point for tumor immunotherapy. However, the most effective way to treat tumors with DCs is the DC vaccine, but the effect is not satisfactory due to the current limitations. At the same time, there are ways to combine DC vaccines with other drugs or cytokines for cancer treatment. For example, in patients with advanced liver cancer, the combined use of DC vaccine and supportive therapy has a lower tumor burden in comparison with patients with supportive therapy alone; meanwhile, the proportion of CD8^+^ T cells is increased, accompanied by a decreased the level of TGF-β (149). For melanoma, the DC vaccine is a promising treatment. Compared with patients treated with the DC vaccine alone, the combination of high-dose systemic IFN-α2b and DC vaccine can significantly prolong overall survival and progression-free survival of melanoma patients (150). Perhaps in future clinical practice, a tailored DC vaccine for each tumor will be a reality. In breast cancer, the combination of statins and cytokines can promote tumor cell apoptosis by targeting the K-Ras on the membrane. Compared with simvastatin alone, human epidermal growth factor receptor-2 (HER2) peptide-pulsed DCs combined with simvastatin has better anti-tumor efficacy, and statin can enhance the anti-tumor function of the corresponding DC vaccine. Using statins reduces the membrane cholesterol content, affecting K-Ras synthesis and further interfering with the downstream PI3K/TBK/AKT intracellular signaling pathway (151). We think that the metabolic regulation in combination with DC vaccines applied in anti-tumor therapy has a significant advantage over DC vaccine usage alone. Unfortunately, there is no effective way to modify DC metabolism so far. But there is great potential in this direction. Future studies in DC metabolism may provide new insights into the more effective treatment of tumors.

## Author Contributions

SL, YT, and XP completed the conception and design of the manuscript. SL, YH, and XP were responsible for writing and/or revising the manuscript. And the guidance of the whole work was mainly completed by SL, YT, and JH. All authors contributed to the article and approved the submitted version.

## Conflict of Interest

The authors declare that the research was conducted in the absence of any commercial or financial relationships that could be construed as a potential conflict of interest.
